# Siphonaxanthin, a Green Algal Carotenoid, as a Novel Functional Compound

**DOI:** 10.3390/md12063660

**Published:** 2014-06-19

**Authors:** Tatsuya Sugawara, Ponesakki Ganesan, Zhuosi Li, Yuki Manabe, Takashi Hirata

**Affiliations:** 1 Division of Applied Biosciences, Graduate School of Agriculture, Kyoto University, Kitashirakawaoiwakecho, Sakyo-ku, Kyoto 606-8502, Japan; E-Mails: ganesan381980@yahoo.com (P.G.); lizhuosi@kais.kyoto-u.ac.jp (Z.L.); yuukim@kais.kyoto-u.ac.jp (Y.M.); hiratan@kais.kyoto-u.ac.jp (T.H.); 2 SRM Research Institute, SRM University, Kattankulathur, Tamilnadu 603 203, India; 3 Department of Rehabilitation, Shijonawate Gakuen University, 5-11-10 Hojo, Daito, Osaka 574-0011, Japan

**Keywords:** angiogenesis, apoptosis, carotenoid, inflammation, green algae, siphonaxanthin

## Abstract

Siphonaxanthin is a specific keto-carotenoid in green algae whose bio-functional properties are yet to be identified. This review focuses on siphonaxanthin as a bioactive compound and outlines the evidence associated with functionality. Siphonaxanthin has been reported to potently inhibit the viability of human leukemia HL-60 cells via induction of apoptosis. In comparison with fucoxanthin, siphonaxanthin markedly reduced cell viability as early as 6 h after treatment. The cellular uptake of siphonaxanthin was 2-fold higher than fucoxanthin. It has been proposed that siphonaxanthin possesses significant anti-angiogenic activity in studies using human umbilical vein endothelial cells and rat aortic ring. The results of these studies suggested that the anti-angiogenic effect of siphonaxanthin is due to the down-regulation of signal transduction by fibroblast growth factor receptor-1 in vascular endothelial cells. Siphonaxanthin also exhibited inhibitory effects on antigen-induced degranulation of mast cells. These findings open up new avenues for future research on siphonaxanthin as a bioactive compound, and additional investigation, especially *in vivo* studies, are required to validate these findings. In addition, further studies are needed to determine its bioavailability and metabolic fate.

## 1. Introduction

Marine algae are a potential renewable resource in the marine environment that are used as a culinary item in East Asia and they have been reported to confer several beneficial effects in human health. They are an excellent source of nutritional and bioactive compounds, such as carotenoids, dietary fibers, amino acids, essential fatty acids, vitamins, and minerals [1,2]. Algal carotenoids have received much attention, as they are structurally different from those found in terrestrial plants. It is important to characterize the novel bio-functional activities of carotenoids from marine algae. 

**Figure 1:**
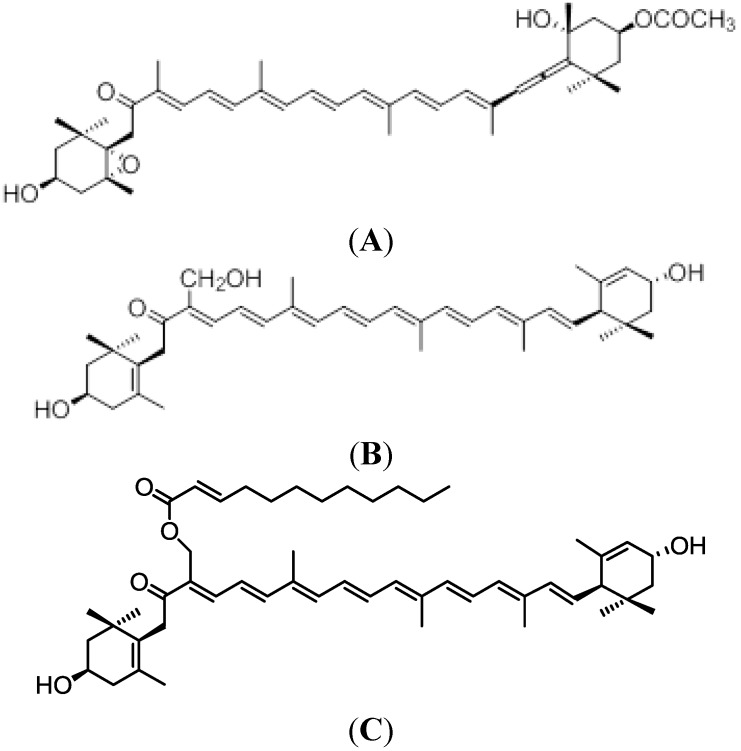
Structures of fucoxanthin (**A**); siphonaxanthin (**B**) and siphonein (**C**).

One of the major carotenoids in marine algae is fucoxanthin, and it is found mainly in brown macroalgae and in some classes of microalgae [3,4,5]. The chemical structure of fucoxanthin includes an allenic bond and oxygenic functional groups, such as hydroxyl, epoxy, carbonyl, and acetyl groups, in addition to its polyene chain (Figure 1A). Recently, fucoxanthin has been reported to possess several beneficial effects related to its anti-cancerous, anti-oxidative, and anti-obesity properties [6,7,8,9,10,11,12]. Moreover, fucoxanthin induces uncoupling protein 1 in the mitochondria of abdominal white adipose tissue, leading to the oxidation of fatty acids and heat production [13]. On the other hand, we found that fucoxanthin and its deacetylated product, fucoxanthinol, effectively suppress angiogenesis [14]. This implies that fucoxanthin, with its anti-angiogenic activity, would be useful in preventing angiogenesis-related diseases such as cancer and diabetic retinopathy. Consequently, brown algal fucoxanthin seems to be a useful bioactive and nutraceutical compound for human health.

Scientific evidence on the bio-functional properties of algal carotenoids is still very limited. Siphonaxanthin (Figure 1B) is a specific keto-carotenoid of green algae, and its bio-functional properties are yet to be clarified. We focus on the potential of siphonaxanthin as a bioactive compound and outline the evidence associated with functionality functionality. The objective of this review is to summarize the novel bio-functional activities of green algal siphonaxanthin that have been highlighted in our studies.

## 2. Siphonaxanthin in Green Algae

Siphonaxanthin is a specific keto-carotenoid of siphonaceous green algae, which helps in absorbing available green and blue green light under water [15,16]. Brown algal fucoxanthin has epoxide and an allenic bond in its structure, whereas siphonaxanthin does not contain either of those functional groups. However, it possesses an additional hydroxyl group on the 19th carbon atom (Figure 1B). In edible green algae such as *Codium fragile*, *Caulerpa lentillifera*, and *Umbraulva japonica*, siphonaxanthin content is approximately 0.03%–0.1% of the dry weight. Siphonein, identified as siphonaxanthin 19-(*trans*-∆2-dodecenoate), is present at the same level as siphonaxanthin in these algae. *C. fragile* is consumed as a part of the staple diet in ancient Japanese food culture. 

Generally, carotenoids in plants play important roles in the photosynthetic process, such as light-absorption and quenching of excess energy [17]. β-Carotene is present in most of the divisions of the reaction-center complexes (RC) and the light-harvesting complexes (LHC) of photosystem I (PSI). On the other hand, in the peripheral LHC of photosystem II (PSII), the bound carotenoids are heterogenous, depending on the classes [5]. In green algae, the major carotenoid in the peripheral LHC of PSII is siphonaxanthin, which exhibited an additional absorption band (the 535 nm band) in the blue to green region. β-Carotene in both RC and LHC-PSI might have protective functions, and carotenoids in the peripheral LHC of PSII might have primarily light-harvesting functions. Employing femtosecond time-resolved fluorescence spectroscopy to analyze purified carotenoids in organic solvents and the LHC in solution, this keto-carotenoid was found to facilitate highly efficient energy transfer of carotenoids to chlorophylls [18]. Siphonaxanthin might have a largely light-harvesting function in the green light-rich underwater habitat [19].

## 3. Apoptosis-Inducing Effect

There is a wealth of information pertaining to apoptosis in anti-cancer research, and the apoptosis-inducing properties of carotenoids may be an important approach to chemo-prevention and/or chemotherapy. Carotenoids from terrestrial plants, such as β-carotene and lycopene, have been extensively studied and implicated as cancer preventive agents. Carotenoids from marine sources have received much attention, as they are structurally different from those found in terrestrial sources. Recent studies have found that carotenoids from algae, including fucoxanthin and peridinin, induce apoptosis in cancer cells [6,7,8,9,20,21]. 

We investigated whether various carotenoids present in marine sources are potentially involved in cancer-preventing action in human leukemia HL-60 cells. We found that the 11 carotenoids investigated, siphonaxanthin most potently at inhibiting the viability of HL-60 cells [22]. Compared to fucoxanthin, siphonaxanthin, at a concentration of 20 μM, markedly reduced cell viability as early as 6 h after treatment. The cellular uptake of siphonaxanthin was 2-fold higher than fucoxanthin, demonstrating a positive correlation between cellular uptake and cell viability. The apoptotic activity of siphonaxanthin was characterized by increases in TUNEL-positive cells, and by increased chromatin condensation in the cells. This induction of apoptosis was accompanied by decreased expression of Bcl-2, and subsequent increase in the activation of caspase-3. These observations indicate that siphonaxanthin may be a more potent growth-inhibitor in cancer cells than fucoxanthin, possibly due to the differences in the structure of their different functional groups. The presence of hydroxyl group on the 19th carbon atom appeared to contribute to the strong apoptosis-inducing effect. This is further supported by the fact that siphonein (Figure 1C), an esterified form of siphonaxanthin that does not contain an additional hydroxyl group, had a reduced inhibitory effect on cell viability. 

Interestingly, siphonaxanthin has been reported to up-regulate the expression of death receptor 5 (DR5) [22]. Tumor necrosis factor-related apoptosis-inducing ligand (TRAIL) promotes apoptosis selectively in tumor cells without much effect on normal cells by binding to the transmembrane receptors TRAIL-R1/DR4 and TRAIL-R2/DR5 [23]. Thus, any chemotherapeutic agent that activates TRAIL-induced apoptosis in cancer cells may be an attractive strategy in anti-cancer research. Yoshida *et al.* reported that halocynthiaxanthin and peridinin sensitize cancer cells to TRAIL-induced apoptosis by up-regulating the expression of DR5 [21], which indicates that some carotenoids can induce apoptosis through this pathway. In our study, siphonaxanthin potently up-regulated the expression of DR5, but fucoxanthin did not [22]. Siphonaxanthin could be potentially useful as a chemopreventive and/or chemotherapeutic agent.

## 4. Anti-Angiogenic Effect

Angiogenesis is characterized by the growth and remodeling process of the primitive network of blood vessels into a complex network. In this process, endothelial cells secrete proteases, migrate through the extracellular matrix, proliferate, and differentiate [24]. Angiogenesis is involved in many physiological and pathological situations. In normal adults, most vasculature is quiescent, with only 0.01% of endothelial cells undergoing division [25]. However, angiogenesis is an essential process in the female reproductive cycle, along with the remodeling and regeneration of tissues [26]. Pathological angiogenesis is implicated in the pathogenesis of many diseases, including cancer, atherosclerosis, diabetic retinopathy, and rheumatoid arthritis [27]. The newly-formed blood vessels promote cancer growth by supplying nutrients and oxygen and by removing waste products. Metastasis also depends on angiogenesis, as tumor cells are shed from a primary tumor and grow in their target organs [28]. Angiogenesis is activated under other pathological conditions, such as ocular and inflammatory disorders [29]. Hence, prevention of angiogenesis under pathological conditions is a promising approach in the prevention of cancer and other pro-angiogenic diseases. It has been reported that some natural products, such as vitamin B_6_ [30], algal polysaccharides [31], and nasunin [32], suppress angiogenesis. Given this background, studies on natural bioactive molecules from marine algae have reported that fucoidans, polysaccharides from marine brown algae, and fucoxanthin and its deacetylated product, fucoxanthinol, exert anti-angiogenic properties [14,33].

To evaluate the anti-angiogenic effect of siphonaxanthin from green algae, we examined its anti-angiogenic effect in cell culture model systems and by employing *ex vivo* approaches using human umbilical vein endothelial cells (HUVECs) and rat aortic ring [34,35], respectively. Siphonaxanthin significantly suppressed HUVEC proliferation at relatively lower concentration of 2.5 µM, while its effect on chemotaxis was not significant [36]. In addition, siphonaxanthin exhibited a strong inhibitory effect on HUVEC tube formation in an *in vitro* angiogenesis model. Siphonaxanthin suppressed the tube length at a concentration of 10 µM, while no tube formation was observed at 25 µM, suggesting that this could be due to the suppression of angiogenic mediators. The *ex vivo* angiogenesis assay using rat aortic ring indicated reduced microvessel outgrowth in a dose-dependent manner, and the reduction was significant at concentrations of more than 2.5 µM. 

To elucidate the molecular mechanism underlying the anti-angiogenic activity of siphonaxanthin compared to fucoxanthin, we focused on the vascular specific pro-angiogenic factor, vascular endothelial growth factor (VEGF), and the other angiogenic factors, such as fibroblast growth factors (FGFs) [37,38]. It has been shown that both siphonaxanthin and fucoxanthin suppress the mRNA expression of fibroblast growth factor 2 (FGF-2) and its receptor (FGFR-1), as well as their *trans*-activation factor, EGR-1 [39]. These suppressive effects of siphonaxanthin were more effective than fucoxanthin. However, the mRNA expression of VEGFR-2, a potent signal transducer involved in the VEGF-mediated signaling pathway, was not significantly affected by these carotenoids. Furthermore, these two marine algal carotenoids can down-regulate the phosphorylation of FGF-2-mediated intracellular signaling proteins such as ERK1/2 and Akt. Inhibition of FGF-2-mediated intracellular signaling proteins by these carotenoids represses the migration of endothelial cells, as well as their differentiation into tube-like structures on Matrigel. These results demonstrate, for the first time, the possible molecular mechanism underlying the anti-angiogenic effect of these two algal carotenoids and suggest that their anti-angiogenic effect is due to the down-regulation of signal transduction by FGFR-1 in vascular endothelial cells. Siphonaxanthin exhibited its anti-angiogenic effect at lower concentrations than fucoxanthin, indicating its potential as a strong angiogenesis inhibitor. These findings provide new insights into the novel bio-functional property of marine algal carotenoids, especially that of siphonaxanthin, which has the potential to enhance current anti-angiogenic therapies in the treatment of cancer and other pro-angiogenic diseases.

## 5. Anti-Inflammatory Effect

Currently, one of the most common social problems in the world is an increasing number of patients with type I allergy. Mast cells play pivotal roles in localized inflammation and immediate type allergic reactions by secreting biologically active substances including histamine, eicosanoids, proteolytic enzymes, cytokines, and chemokines after antigen-induced degranulation. The antigen-induced aggregation of the high affinity IgE receptor (FcεRI) expressed on the cell surface triggers the degranulation of mast cells [40]. We have previously reported that astaxanthin, β-carotene, fucoxanthin, and zeaxanthin significantly inhibit antigen-induced degranulation of rat basophilic leukemia RBL-2H3 cells, which were used as a mast cell model, and bone marrow-derived mast cells [41]. Interestingly, these carotenoids inhibited antigen-induced translocation of FcεRI to lipid rafts, which are known as platforms of the aggregation of FcεRI [42]. Furthermore, oral administration of these four carotenoids for a week significantly inhibited dinitrofluorobenzene (DNFB)-induced ear swelling and the increased content of histamine in the DNFB-treated mice [43]. These results suggested that dietary carotenoids exert an anti-inflammatory effect by suppressing mast cell degranulation *in vivo*. However, information about the anti-degranulation effect of the other carotenoids is limited.

In this context, we evaluated the effects of eleven additional carotenoids using the RBL-2H3 cells. Results from our screening showed that nine carotenoids, including siphonaxanthin, had inhibitory effects on antigen-induced degranulation of mast cells [44]. The inhibitory activity of carotenoids was not related to their cellular uptake. It is speculated that carotenoids, including siphonaxanthin, may modify the functions of lipid rafts by localizing in the cell membrane and inhibiting the translocation of FcεRI to lipid rafts. Nevertheless, it is important to verify whether carotenoids affect other signaling pathways involved in lipid rafts in order to understand the biological relationship between carotenoids and lipid rafts.

## 6. Conclusions and Future Perspectives

To clarify the biological action of siphonaxanthin, further studies are required to determine its bioavailability and biological metabolism. Our previous studies, using cultured cells and mice, demonstrated that orally administered fucoxanthin is metabolized to fucoxanthinol and amarouciaxanthin A in the intestinal tract and liver, respectively [45,46]. However, information on the bioavailability and metabolic conversion of siphonaxanthin *in vivo* are limited. Siphonein, which is identified as siphonaxanthin 19-(trans-∆2-dodecenoate) is present in similar levels as that of siphonaxanthin in siphonaceous green algae. Based on our data using cultured cells and *ex vivo* studies, the biological functions of siphonein were found to be weaker than those of siphonaxanthin. If siphonein could be hydrolyzed in the intestine by digestive enzymes and absorbed in a manner similar to siphonaxanthin, oral administration of siphonein could be expected to exert the same biological activities as siphonaxanthin. 

Siphonaxanthin isolated from marine green algae, such as *C. fragile*, remarkably suppresses cell viability, induces apoptosis in cancer cells, and possesses more potent anti-angiogenic activity than fucoxnathin. Although several reports have indicated that fucoxanthin could be immensely beneficial to human health, our studies indicate that siphonaxanthin is more effective than fucoxanthin. Nevertheless, these findings uncover new avenues for future research on siphonaxanthin as a bioactive compound, and additional investigation, especially *in vivo* studies, are needed to validate the reported findings. 
